# Ultrasound characterization of breast masses

**DOI:** 10.4103/0971-3026.54878

**Published:** 2009-08

**Authors:** Sudheer Gokhale

**Affiliations:** Dr Gokhale's Sonography clinic, Indore, India

**Keywords:** Breast ultrasound, Breast mass, ACR BIRADS-US criteria

## Abstract

A lump in the breast is a cause of great concern. High frequency, high-resolution USG helps in its evaluation. This is exemplified in women with dense breast tissue where USG is useful in detecting small breast cancers that are not seen on mammography. Several studies in the past have addressed the issue of differentiating benign from malignant lesions in the breast. The American College of Radiology has also brought out a BIRADS-US classification system for categorizing focal breast lesions.

## Introduction

Breast cancer is among the most common causes of cancer deaths today, coming fifth after lung, stomach, liver and colon cancers. It is the most common cause of cancer death in women.[1] In 2005 alone, 519 000 deaths were recorded due to breast cancer.[1] This means that one in every 100 deaths worldwide and almost one in every 15 cancer deaths were due to breast cancer. Refinement of high-frequency technology, particularly with 7.5–13 MHz probes, has brought out a totally new facet in USG breast imaging.[2] For example:

- High-density probes provide better lateral resolution
- Harmonic imaging leads to improved resolution and reduced reverberation and near-field artifacts
- Real-time compound scanning results in increased tissue contrast resolution
- Extended or panoramic views provide a better perspective of the lesion in relation to the rest of the breast

Harmonic imaging and real-time compounding has been shown to improve image resolution and lesion characterization.[3,4] More recently, USG elastography seems to be quite promising. Initial results indicate that it can improve the specificity and positive predictive value of USG in the characterization of breast masses.[5]

The reason why any lesion is visible on mammography or USG is the relative difference in the density and acoustic impedance of the lesion, respectively, as compared to the surrounding breast tissue.

This is exemplified in women with dense breast tissue, where USG is useful in detecting small breast cancers that are not detected on mammography.[6]

## Normal breast parenchymal patterns

**Figure 1:**
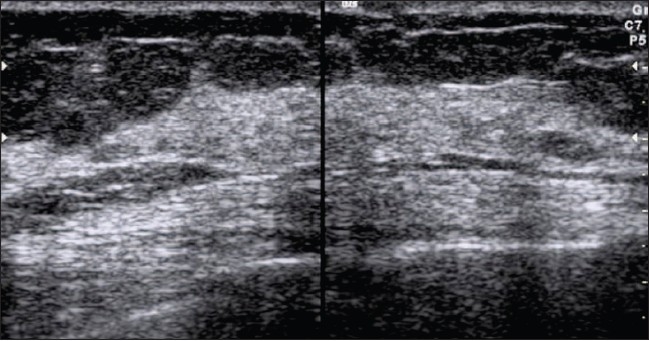
Normal breast. Mid transverse scan of a normal breast. The fibroglandular parenchyma is echogenic (arrowheads) and is surrounded by hypoechoic fat (*)

In the young non-lactating breast, the parenchyma is primarily composed of fibroglandular tissue, with little or no subcutaneous fat. With increasing age and parity, more and more fat gets deposited in both the subcutaneous and retromammary layers[7] [Figure 1].

## Abnormal appearances

### Breast cysts

**Figure 2 (A–D):**
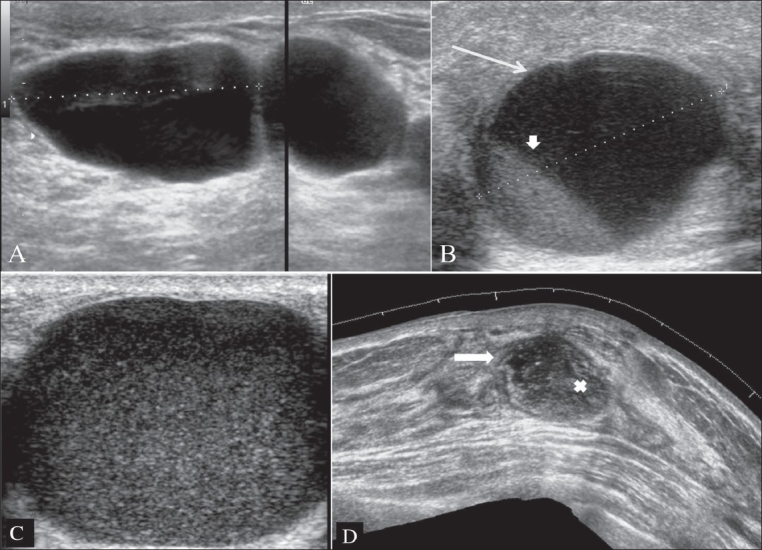
Cysts. Cysts usually reveal thin walls and through transmission (A). An inflamed cyst (B) reveals A thick edematous wall (arrow) with internal layering of thick/thin fluid (arrowhead). A galactocele (C) reveals diffuse low-level echoes in the cyst. chronic abscess (D) seen in this extended views shows an an irregular pseudo-wall (arrow) with dirty internal echoes due to pus or debris (X).

Breast cysts are the commonest cause of breast lumps in women between 35 and 50 years of age.[7] A cyst occurs when fluid accumulates due to obstruction of the extralobular terminal ducts, either due to fibrosis or because of intraductal epithelial proliferation. A cyst is seen on USG as a well-defined, round or oval, anechoic structure with a thin wall [Figure 2A]. They may be solitary or multiple [Figure 2B].

*Complex cyst*: When internal echoes or debris are seen, the cyst is called a complex cyst. These internal echoes may be caused by floating cholesterol crystals, pus, blood or milk of calcium crystals.[8] [Figure 2C].

### Chronic abscess of the breast

Patients may present with fever, pain, tenderness to touch and increased white cell count. Abscesses are most commonly located in the central or subareolar area.[9] An abscess may show an ill-defined or a well-defined outline. It may be anechoic or may reveal low-level internal echoes and posterior enhancement [Figure 2D].

### Fibrocystic breast condition

**Figure 3 (A-C):**
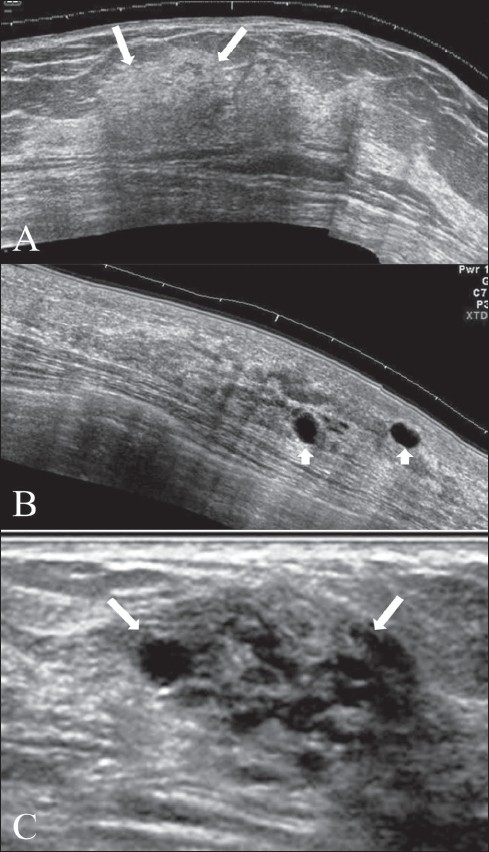
Fibrocystic change. Extended view images (A, B) show a focal area of thickening of the breast parenchyma (A) with patchy increase in echogenicity (arrows) and scattered, discrete, thin-walled cysts (arrowheads in B). The “lump” may shows a combination of clustered tiny cysts and thickened parenchyma (arrows in C)

This condition is referred to by many different names: fibrocystic disease, fibrocystic change, cystic disease, chronic cystic mastitis or mammary dysphasia. The USG appearance of the breast in this condition is extremely variable since it depends on the stage and extent of morphological changes. In the early stages, the USG appearance may be normal, even though lumps may be palpable on clinical examination. There may be focal areas of thickening of the parenchyma, with or without patchy increase in echogenicity [Figure 3A]. Discrete single cysts or clusters of small cysts may be seen in some [Figure 3B and C]. Focal fibrocystic changes may appear as solid masses or thin-walled cysts. About half of these solid masses are usually classified as indeterminate and will eventually require a biopsy.[10]

### Duct ectasia

**Figure 4 (A, B):**
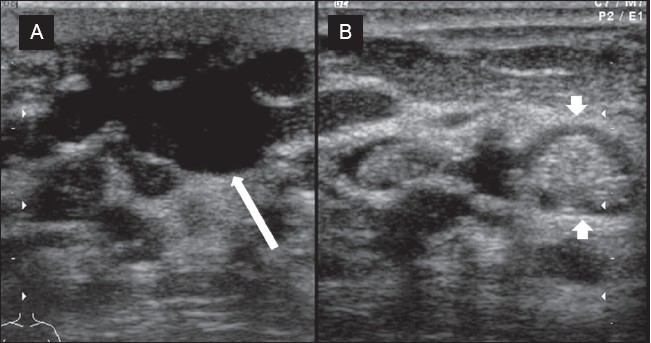
Chronic duct ectasia. Longitudinal image (A) shows a dilated duct containing inspissated debris (arrow) is seen. In crosssection (B), the intraductal debris may appear as a focal lesion (arrowheads)

This lesion has a variable appearance. Typically, duct ectasia may appear as a single tubular structure filled with fluid or sometimes may show multiple such structures as well. Old cellular debris may appear as echogenic content. If the debris fills the lumen, it can be sometimes mistaken for a solid mass, unless the tubular shape is picked up[11] [Figure 4].

### Fibroadenoma

**Figure 5:**
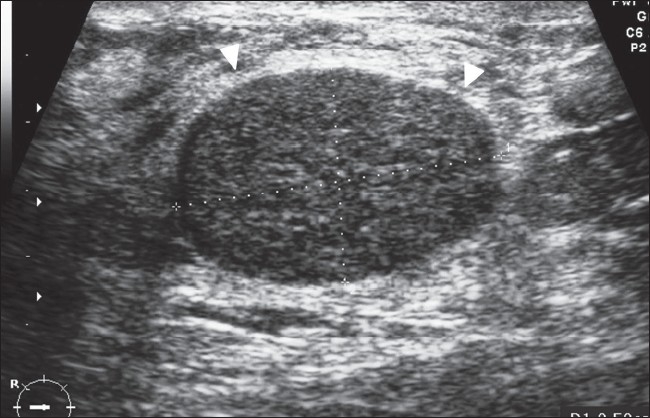
Fibroadenoma. Transvere image reveals a typical larger transverse than anteroposterior diameter, homogenous echotexture, and a thin capsule (arrowheads)

Fibroadenoma is an estrogen-induced tumor that forms in adolescence. It is the third most common breast lesion after fibrocystic disease and carcinoma. It usually presents as a firm, smooth, oval-shaped, freely movable mass.[12] It is rarely tender or painful. The size is usually under 5 cm, though larger fibroadenomas are known. Fibroadenomas are multiple in 10–20% and bilateral in 4% of cases. Calcifications may occur. On USG, it appears as a well-defined lesion [Figure 5]. A capsule can usually be identified. The echotexture is usually homogenous and hypoechoic as compared to the breast parenchyma, and there may be low-level internal echoes. Typically, the transverse diameter is greater than the anteroposterior diameter [Figure 5]. In a small number of patients, the mass may appear complex, hyperechoic or isoechoic. A similar USG appearance may be seen with medullary, mucinous or papillary carcinoma.[13]

### Cystosarcoma phyllodes

**Figure 6:**
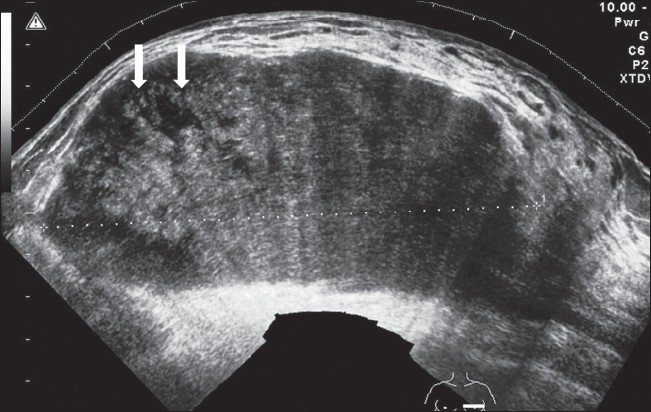
Cystosarcoma phyllodes. Transverse scan reveals a large well-defined mass. There is inhomogeneous echotexture, with small areas of cystic degeneration (arrows)

This is a large lesion that presents in older women. Some authors consider it to be a giant fibroadenoma. The mass may involve the whole of the breast. It usually reveals well-defined margins and an inhomogeneous echostructure, sometimes with variable cystic areas. The incidence of malignant change is low. [7] [Figure 6B].

### Lipoma

**Figure 7:**
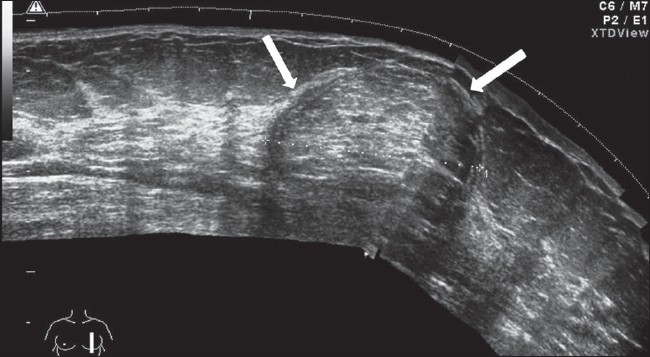
Lipoma. Sagittal extended view reveals a subtle echogenic mass with a reticular pattern and a well-defined, thin capsule (arrows)

Lipoma is a slow-growing, well-defined tumor. It may be a chance finding or the patient may present with complaints of increase in the size of the involved breast, though no discretely palpable mass can be made out. The tumor is soft and can be deformed by compression with the transducer. A thin capsule can usually be identified and the tumor often reveals an echogenic structure, with a stippled or lamellar appearance[13] [Figure 7A and B].

### Breast ultrasound: criteria for benign lesions

Several studies have described the sonographic characteristics commonly seen in benign lesions of the breast:[14,15]

1. Smooth and well circumscribed
2. Hyperechoic, isoechoic or mildly hypoechoic
3. Thin echogenic capsule
4. Ellipsoid shape, with the maximum diameter being in the transverse plane
5. Three or fewer gentle lobulations
6. Absence of any malignant findings

## Characteristics of malignant lesions

**Figure 8 (A–F):**
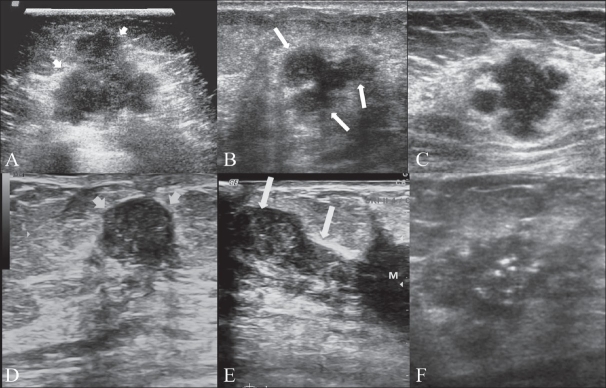
Malignant lesions. Transverse scan (A) shows a typical malignant nodule that is taller than wide, with hypoechoic echotexture. Arrowheads indicate irregular spiculated margins. Some of the nodules may reveal a branching pattern (arrows in B). Sagittal view (C) shows a nodule with multilobulated margins; the presence of more than 3–4 lobulations is suspicious for malignancy. Sagittal (D) and transverse (E) scans show duct extension (arrows). ‘M’ indicates the primary site. Duct extension appears smooth in outline in cross-section (arrowheads in E). Transverse scan (F) shows a typical malignant lesion with irregular spiky margins, microcalcifications and a branching pattern. This lesion is classifiable as US-BIRADS category 4

**Figure 9 (A,B):**
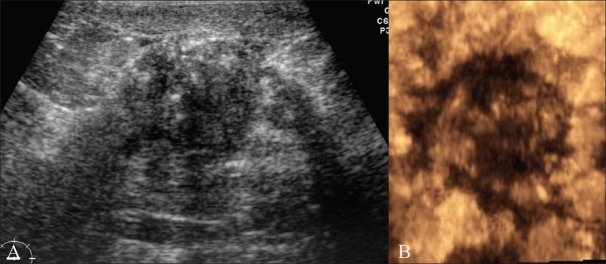
Malignant lesion. Transverse scan (A) shows smooth margins, suggesting a category 3 lesion. A 3Dimage in the coronal plane (B) however reveals spiky margins with a sunray appearance, suggestive of a category 4 lesion

**Figure 10:**
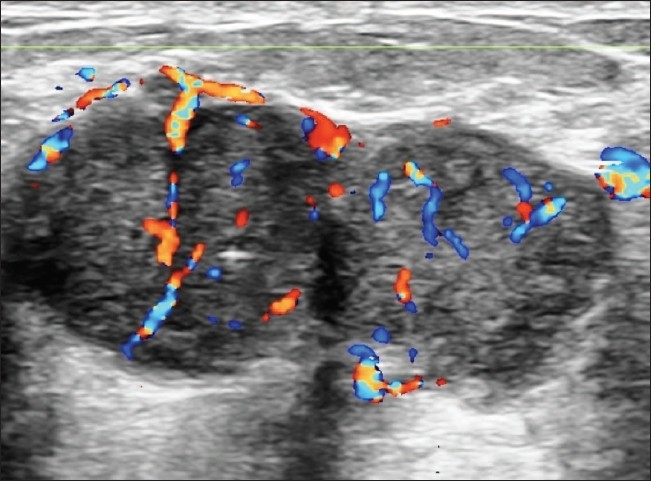
Malignant lesion. A smooth margin and homogenous echotexture suggest a category 3 lesion. Color Doppler reveals irregularly branching neovascularity

Malignant lesions are commonly hypoechoic lesions with ill-defined borders. Typically, a malignant lesion presents as a hypoechoic nodular lesion, which is ‘taller than broader’ and has spiculated margins, posterior acoustic shadowing and microcalcifications[13] [Figure 8A–F]. Three-dimensional scanners with the capability of reproducing high-resolution images in the coronal plane provide additional important information. The spiky extensions along the tissue planes can be well seen in coronal images[16] [Figures 9A and B]. It was initially believed that color Doppler scanning would add to the specificity of USG examination, but this has not proven to be very efficacious; however, in certain situations it does help resolve the issue, particularly when there is significant vascularity present within highly cellular types of malignancies[17] [Figure 10].

In a landmark study in 1995, Stavros *et al*. established USG criteria to characterize solid breast masses [Table 1].[14]

**Table 1:** USG suspicious for malignancy

Finding: Solid nodule	Positive predictive value
Spiculation	91.8
Taller than wide	81.2
Angular margins	67.5
Shadowing	64.9
Branching pattern	64.0
Hypoechogenicity	60.1
Calcifications	59.6
Duct extension	50.8
Branching pattern	48.0
Microlobulations	48.2

## Discussion

Although it may be impossible to distinguish all benign from all malignant solid breast nodules using USG criteria, a reasonable goal for breast USG is to identify a subgroup of solid nodules that has such a low risk of being malignant that the option of short-interval follow-up can be offered as a viable alternative to biopsy. In a 4-year follow-up of palpable, circumscribed, noncalcified solid breast masses (similar to BI-RADS category 3), Graf *et al*. found that such cases can be adequately managed with short-term follow-up at 6-month intervals for 2 years.[18]

Combined studies, which included USG and mammography, have demonstrated a near 100% negative predictive value for palpable breast lesions, when both are used together.[19,20]

In a study based on characterization of breast masses according to BIRADS-US criteria, Kwak *et al*. found no statistical differences between fine-needle aspiration cytology and USG with regard to sensitivity and Negative Predictive value (*P* > 0.05).[21] Heinig *et al*. also found USG characterization of breast lesions using BIRADS-US criteria to be highly accurate.[22]
